# Pathways to Health: Association Between Trail Use, Weight Status, and Self-Rated Health Among Adults in Greenville County, South Carolina, 2014

**DOI:** 10.5888/pcd13.160197

**Published:** 2016-12-15

**Authors:** S. Morgan Hughey, Andrew T. Kaczynski, Morgan N. Clennin, Julian A. Reed

**Affiliations:** 1 University of South Carolina, Arnold School of Public Health, Department of Health Promotion, Education, and Behavior, Columbia, South Carolina; 2 University of South Carolina, Prevention Research Center, Columbia, South Carolina; 3 University of South Carolina, Arnold School of Public Health, Department of Exercise Science, Columbia, South Carolina; 4 Furman University, Department of Health Sciences, Greenville, South Carolina.

## Abstract

We examined associations between adults’ use of a prominent rail-trail and their weight status and self-rated health. In 2014, a random-digit-dial survey of Greenville County, South Carolina, residents (n = 639) was used to collect data on trail use, height and weight, self-rated health, and demographics. Trail users were half as likely to be overweight or obese as trail nonusers (odds ratio [OR] = 0.56; 95% confidence interval [CI], 0.33–0.95). Similarly, trail users were significantly more likely to report high self-rated health than were trail nonusers (OR = 1.83; 95% CI, 1.13–2.97). Findings suggest that trail use is associated with healthier weight status and higher self-rated health and supports the development, maintenance, and promotion of trail resources.

## Objectives

Trails are important community features for encouraging active recreation and transportation, connecting residents with destinations (eg, parks, retailers), and supporting economic development (1–3). One review documented that prospective and correlational studies report mixed findings, including positive associations between trails and various active behaviors, but cautioned that more evidence was needed (1). Although much research has examined relationships between trails and physical activity (PA), few studies have explored the association between trail use and health outcomes (4). Measures of adult weight status and self-rated health are consistently predictors of morbidity and mortality rates among diverse populations (5). The purpose of this study was to examine associations between trail use and weight status and self-rated health.

## Methods

Data were collected in March 2014 through a random-digit–dial survey in Greenville County, South Carolina. The Greenville Health System Swamp Rabbit Trail (GHSSRT) is a 21-mile paved rail-trail and an important component of the county’s infrastructure; approximately 43,000 residents live within 1 mile of trail access points. The survey was conducted with adults aged 18 years or older (response rate = 28.0%) and was adapted from an intercept survey developed to assess trail-related behaviors and perceptions as well as respondent demographics and health-related outcomes (7); the random-digit–dial methods of the survey are described elsewhere (6). Furman University’s institutional review board approved this study.

The main independent variable for this study was trail use, which was measured by asking respondents whether they used the GHSSRT in the previous 6 months (ie, September 2013 through February 2014, yes or no). Participants reported their height and weight, and their body mass index (BMI, kg/m^2^) was calculated and categorized as normal weight (BMI = 18.0–24.9) or overweight or obese (BMI ≥25.0) (8). Self-rated health was measured by asking, “Compared to other people your age, would you say your overall health is poor, fair, good, very good, or excellent?”; responses were categorized as low (≤3.0) or high (>3.0), on the basis of the median value (3.0) (5).

Finally, the survey assessed 4 demographic characteristics: sex (male/female), age (18–64 or ≥65 y), education (high school diploma or less, some college or college degree, and greater than college degree), and race/ethnicity (eventually grouped as nonwhite vs non-Hispanic white). Distance from the respondent’s address to the nearest trail access point was calculated using ArcGIS 10.2.2 (Esri).

Logistic regression was used to assess associations between trail use and weight status (overweight/obese vs normal weight) as well as self-rated health (low vs high). Model 1 examined unadjusted estimates between trail use and both dependent variables, and Model 2 examined estimates after controlling for all participant demographics and residential distance from the trail, which are associated with PA, obesity and self-rated health (9,10). We analyzed the data using Stata version 13.0 (StataCorp LP) and included data from all participants for whom we had complete data on all variables (n = 408 for weight status, n = 506 for self-rated health). No significant differences in weight status or self-rated health were observed between participants in the final samples and participants for whom data on some covariates were missing.

## Results

Most of the sample was female (58.3%), aged 18 to 64 years (52.3%), non-Hispanic white (87.2%), and had greater than a high school diploma (71.0%) (Table 1). Approximately 60% of participants were overweight or obese, and about half (49.2%) reported high self-rated health. Three-quarters (75.1%) of the sample reported not using the trail during the previous 6 months. Men were more than twice as likely as women to be overweight or obese, and higher levels of education were associated with higher self-rated health (Table 2).

**Table 1:** Demographic Characteristics of Participants, Study on Associations Between Trail Use, Weight Status, and Self-Rated Health, Greenville County, South Carolina, 2014^a^

Characteristic	Total Sample, n = 639	Trail Users, n = 159	Trail Nonusers, n = 480
**Body mass index (kg/m^2^)**			
Normal weight (18.0–24.9)	41.5	52.6	37.6
Overweight or obese (≥25.0)	58.5	47.4	62.4
**Self-rated health^b^**			
Low	50.8	33.6	56.7
High	49.2	66.4	43.3
**Sex**			
Female	58.3	51.7	60.6
Male	41.7	48.3	39.4
**Age, y**			
18–64	52.3	71.0	46.0
≥65	47.7	29.3	54.0
**Race**			
Nonwhite	12.8	11.2	13.4
Non-Hispanic white	87.2	88.8	86.6
**Education status**			
≤High school diploma	29.0	34.5	13.5
Some college or college degree	52.7	51.0	57.4
>College degree	18.3	14.6	29.1
**Mean distance from respondents’ address to trail, mi (standard deviation)**	8.1 (4.9)	8.2 (5.8)	8.1 (4.7)

a Values expressed as percentages, unless otherwise indicated.

b Self-rated health was measured by asking, “Compared to other people your age, would you say your overall health is poor, fair, good, very good, or excellent?”; responses were categorized as low (≤3.0) or high (>3.0), on the basis of the median value (3.0).

**Table 2:** Association of Trail Use With Weight Status and Self-Rated Health Among Adults, Greenville County, South Carolina, 2014

Characteristic	Overweight/Obese, n = 408		High Self-Rated Health^a^, n = 506	
	Model 1	Model 2	Model 1	Model 2
	Odds Ratio (95% Confidence Interval)			
**Trail use**				
No	1 [Reference]			
Yes	0.54 (0.32–0.83)^b^	0.56 (0.33–0.95)^b^	2.48 (1.70–3.63)^b^	1.83 (1.13–2.97)^b^
**Sex**				
Female	1 [Reference]			
Male	—	2.47 (1.59–3.83)^b^	—	1.08 (0.74–1.58)
**Age, y**				
18–64	1 [Reference]			
≥65	—	1.00 (0.65–1.51)	—	0.85 (0.59–1.23)
**Race**				
Nonwhite	1 [Reference]			
Non-Hispanic white	—	0.52 (0.27–1.03)	—	1.67 (0.94–2.97)
**Education**				
≤High school degree	1 [Reference]			
Some college or college degree	—	0.63 (0.39–1.02)	—	2.24 (1.46–3.45)^b^
>College degree	—	0.63 (0.33–1.20)	—	2.59 (1.47–4.56)^b^
**Distance of respondent’s address from trail**	—	1.02 (0.97–1.06)	—	0.99 (0.95–1.02)

a Self-rated health was measured by asking, “Compared to other people your age, would you say your overall health is poor, fair, good, very good, or excellent?”; responses were categorized as low (≤3.0) or high (>3.0), on the basis of the median value (3.0).

b *P* < .05.

After controlling for all covariates, trail users were significantly less likely to be overweight or obese compared with trail nonusers (odds ratio [OR] = 0.56; 95% confidence interval [CI], 0.33–0.95) (Table 2). Additionally, trail users were significantly more likely to report high self-rated health than were trail nonusers (OR = 1.83; 95% CI, 1.13–2.97).

## Discussion

Our findings suggest that trail use is associated with healthier weight status and higher self-rated health. Several mechanisms may explain the observed associations. First, trails are associated with meeting PA recommendations, which has substantial health benefits (11), and they facilitate diverse modes of PA, including walking and cycling. Second, trails often offer an aesthetically pleasing natural environment in which to be active. For example, much of the GHSSRT runs parallel to the Reedy River, a water feature that may enhance users’ experiences, and aesthetics/scenic beauty was the second-highest reported reason for using the trail among survey respondents (22%). Such green spaces are also linked to reduced stress and improved mood (12). Lastly, community trails like the GHSSRT may encourage increased social connections and sense of community. Since trail construction, small businesses and community events have grown substantially along the GHSSRT. Overall, trails are vital community resources for promoting active and healthy communities through a variety of physical, psychological, and social mechanisms.

Several limitations to this study should be noted. First, the study design was cross-sectional, limiting ability to determine causality. Also, the primary variables were self-reported measures of trail use, health status, and height and weight; the telephone survey was based on a sample of residential phone numbers, and the participants were mostly white and educated, which may limit the study’s generalizability. In addition, respondents reported trail use patterns during fall and winter months, which are milder in South Carolina; however, future studies should examine effects of seasonality. Future research should also include longitudinal assessments and dose–response relationships between trail use, PA, weight status, and health outcomes over time, including whether this relationship differs for adults with varying levels of overweight and obesity. In summary, understanding how community resources such as trails are associated with diverse indicators of well-being can lend greater evidence to justify their construction in communities as key pathways to promote health.
